# A study of the context in which compact intracloud discharges occur

**DOI:** 10.1038/s41598-019-48680-6

**Published:** 2019-08-21

**Authors:** Adonis F. R. Leal, Vladimir A. Rakov

**Affiliations:** 1 Department of Electrical and Biomedical Engineering, Federal University of Para (UFPA), Belém, Brazil; 2 Department of Electrical and Computer Engineering, University of Florida (UF), Gainesville, USA; 3 Moscow Institute of Electronics and Mathematics, National University Higher School of Economics, Moscow, Russia

**Keywords:** Electrical and electronic engineering, Characterization and analytical techniques

## Abstract

The occurrence context of compact intracloud discharges (CIDs) is examined using their electric field waveforms and corresponding NLDN data. A total of 1096 CIDs transporting negative charge upward and 8 CIDs transporting positive charge upward were analyzed. The CIDs were categorized based on whether they were isolated or were followed, preceded, or both followed and preceded by other NLDN-reported lightning events. The percentages of isolated CIDs transporting negative charge upward decreased from 92% for 5 km search radius and ±10 ms time window to 31% for 10 km and ±1000 ms, this decrease being accompanied by an increase of the percentage of CIDs preceding (initiating) normal lightning events from 6.8% to 43%. GM NLDN-reported peak currents for isolated CIDs (33 kA) were similar to those initiating normal lightning events (34 kA). Some of our isolated CIDs could be viewed as precursors, because they apparently initiated normal lightning events at essentially the same location after time intervals measured in seconds. CIDs transporting positive charge upward (a) occurred at heights ranging from 16 to 19 km vs. 6 to 16 km for CIDs transporting negative charge upward and (b) had considerably higher NLDN-reported peak currents: 113 kA vs. 33 kA (GM values).

## Introduction

Compact intracloud discharges (CIDs) are lightning discharges inside the cloud that are characterized by short (usually shorter than 1 km) inferred channel lengths, very strong HF-VHF (3–300 MHz) radiation, and characteristic bipolar wideband electric field pulses (referred to as Narrow Bipolar Pulses or NBPs) having a typical total width of 10–30 μs and large amplitudes that are comparable to those of pulses produced by return strokes (RSs) in cloud-to-ground discharges (CGs) at similar distances. At short distances, electromagnetic signatures of CIDs can be dominated by the induction field component and, hence, are not necessary bipolar (e.g., Eack^1^; Karunarathne *et al*.^2^). CIDs are also known as narrow bipolar events (NBEs).

Observations of CIDs were first reported by Le Vine^3^. Since then, many researchers have been reporting this phenomenon from observations in different regions of the world including the United States^4–9^, Sri Lanka^10^, Malaysia^11^, China^12–16^, and Japan^17^.

Compared to other forms of lightning, our understanding of the physics of CIDs is still poor. Gurevich *et al*.^18^ and Gurevich and Zybin^19^ proposed the relativistic runaway electron avalanche as a possible mechanism of CID. Cooray *et al*.^20^ modeled the CID as one or more relativistic avalanches and related the observed “noisiness” of dE/dt waveforms produced by CIDs to multiple avalanche burst. However, Rison *et al*.^21^ argued that there is little or no evidence that energetic electron avalanches are involved in CIDs and proposed their own CID mechanism: extremely fast (>10^7^ m/s) positive breakdown occurring in virgin air and leaving behind no conducting channel. More recently, Tilles *et al*.^22^ reported that a similarly fast breakdown of opposite (negative) polarity can also produce CIDs. Nag and Rakov^23^ observed periodic variation in their dE/dt records of CIDs and inferred that, from the electromagnetics point of view, the CID is essentially a bouncing-wave phenomenon. They suggested that the process could be viewed as a long wave repeatedly folding on itself, putting aside the question of how the wave-guiding structure was created. Rison *et al*.^21^, on the other hand, visualized their fast positive breakdown as a system of streamers with proprieties that are not supported by observations of streamers in laboratory, all of which are indicative of maximum streamer speeds <10^7^ m/s vs. 10^7^ to 10^8^ m/s reported for the fast positive breakdown. There is currently a debate on the role of CIDs in lightning initiation.

A number of researches reported that CIDs tend to be temporally isolated from other lightning events^3–6^. Medelius *et al*.^24^ analyzed 156 negative and 10 positive (atmospheric electricity sign convention) narrow bipolar pulses that occurred during overhead thunderstorms at the Kennedy Space Center, Florida. They reported that about two-thirds of 30 pulses identified in continuous magnetic tape records occurred “more than one second apart from any burst activity typical of lightning”. Nag *et al*.^6^ found that 73% of CIDs (114 of 157) occurred in insolation (77% if 6 isolated CIDs that occurred in pairs are additionally included) in a time window with 100 ms before and 400 ms after the CID, while Smith *et al*.^5^ used relatively narrow (4 to 50 ms) time windows. Some studies have shown that CIDs often immediately precede IC flashes and may initiate them. Weidman and Krider^25^ noted that the first signals detected from developing storms usually were “fast negative-polarity wave forms with relatively short pulse widths”. Bils *et al*.^26^ and Villanueva *et al*.^27^ noted that characteristic CIDs waveforms occurred in the early stage of cloud flashes. Rison *et al*.^28^, who used a three-dimensional VHF (63 MHz) TOA lightning locating system, found that the discharge process giving rise to narrow bipolar pulses was the initial event of an “otherwise normal intracloud discharge”. More recently, Wu *et al*.^29^ found that 103 (16.1%) out of 638 CIDs occurred as initial events of IC lightning flashes. According to Rison *et al*.^21^, many or possibly all lightning flashes are initiated by fast positive breakdown, which, they suggest, is the unique physical process behind CIDs. Most lightning discharges, however, are known to be not preceded by a CID-like electromagnetic field signature (e.g., Nag *et al*.^30^; Marshall *et al*.^31^; Lyu *et al*.^32^). Different lightning initiation scenarios seem to be possible.

Nag *et al*.^6^ reported that only 18% of their CIDs accompanied ordinary IC flashes. In all 8 cases for which NLDN locations were available, CIDs preceded IC impulsive processes, with 7 being within 10 km of ICs. Five CIDs preceded ICs by 5.3–67 ms with horizontal separation distances of 1 km or less. They also reported that 6% of their CIDs were associated with CG flashes. Three out of seven such CIDs located by the NLDN preceded CGs by 72–233 ms, while 4 occurred during or after CG flashes. Five out seven were within 10 km of CGs.

Thus, there appears to be controversy in the literature regarding whether CIDs are rare and isolated events or they are common and serve to initiate other lightning events. The disparity may be in part due to the use of different time windows in studying the CID occurrence context. In this paper, we will use NLDN data to examine association (or lack of association) of CIDs with other lightning events for different search radii and time windows. In case of CIDs accompanied by other lightning events, we will examine the relative intensity (in terms of NLDN-reported currents) of CIDs and their accompanying events. Possibility of viewing CIDs as precursors that initiate normal lightning activity at the same location after time intervals measured in seconds will be examined.

Some researchers (e.g., Willett *et al*.^4^; Wu *et al*.^13^; Karunarathne *et al*.^7^) ascribed polarity (plus or minus sign) to their observed CIDs, based on the adopted electrical field polarity convention (e.g., Rakov and Uman^33^, Section 1.4.2). According to the atmospheric electricity sign convention, CIDs transporting negative charge upward produce electric field pulses with negative initial half-cycle and are labeled as −CIDs, and CIDs transporting positive charge upward produce electric field pulses with positive initial half-cycle and are labeled as +CIDs. If one switches to the physics sign convention, the CID polarity should be reversed (−CID becomes +CID and +CID becomes −CID), which sometimes causes confusion. In our opinion, assigning polarity to CIDs is not justified, because they neutralize equal amounts of positive and negative charge (as any IC discharges), in contrast with CGs whose polarity indicates the sign of charge effectively transported to the ground. In this study, we use the terms “lower-level CID” for CIDs transporting negative charge upward and “upper-level CID” for CIDs transporting positive charge upward. This new labeling is illustrated in the context of typical cloud charge structure in Fig. A1 of this paper. Note that lower-level CIDs are considerably more numerous than upper-level CIDs. In some studies, the latter were not observed at all (e.g., Lu *et al*.^16^). Lower-level CIDs correspond to −CIDs and upper-level CIDs to +CIDs in the atmospheric electricity sign convention.

## Data

CIDs analyzed in this paper were recorded at the Lightning Observatory in Gainesville (LOG), Florida^34^, within a range of 500 km of LOG. Electric field signatures of a total of 1104 CIDs (both lower- and upper-level types) were recorded at distances ranging from 9.3 to 495 km in August 2016. The distances were calculated based on NLDN reported locations. The electric field waveforms (all dominated by the radiation field component) were obtained using the Lightning Detection and Waveform Storage System (LDWSS)^35^. The LDWSS had a bandwidth from 160 Hz to 500 kHz, the decay time constant of 1 ms, and the time resolution (sampling interval) of 1 μs. The LDWSS recorded up to 5 windows of 5 ms each, without dead time, so that the maximum record length was 25 ms. There was a dead time associated with data transfer to the computer-based storage system which is up to 10 s or so, depending on the number of windows transferred. The LDWSS dead time does not affect our results, because we used only those events which were recorded by both LDWSS and NLDN, and the CID context was analyzed based only on the NLDN data that are recorded without any dead time.

The LDWSS was triggered when the electric field exceeded a set threshold. Each time that the threshold was exceeded, the system stored a 5-ms window. Not only CIDs, but also pulses associated with CGs and ordinary ICs were recorded. We distinguished CIDs from other lightning events based on a semi-automated procedure developed by Leal *et al*.^9^. This procedure identified CIDs based on their frequency spectra (within the 160 Hz–500 kHz LDWSS bandwidth) and time-domain waveforms. All the events analyzed in this study were required to be recorded by the U.S. National Lightning Detection Network (NLDN), in order to control the distance and the intensity (represented by NLDN-reported peak current) of the source. Many of CIDs were misclassified by the NLDN as +CGs. More details of the semi-automated procedure and misclassification of CIDs by the NLDN are given by Leal *et al*.^9^. Data from the NLDN were also used to search for and identify other lightning events before and after the CID.

Our results for isolated CIDs depend on NLDN detection efficiency (DE), because some isolated CIDs can result from the NLDN failure to detect both preceding and following events. Since the NLDN DE and classification accuracy (CA) for CG return strokes are each higher than 90% (see the Methods section and ref.^36^ therein), our results on isolation of CIDs from CG strokes should be reliable. Also, CIDs initiating “normal” (complete) IC flashes are unlikely to be misclassified as isolated, because the DE for IC flashes is >70%. In our analyses, we will pay special attention to testing the validity of the claim that CIDs often initiate “normal” ICs. The results of this testing should be also reliable, because 73% of such ICs are expected to be detected by the NLDN, with only 5% being misclassified.

The number of lower-level CIDs recorded in different distance ranges is given in Table 1. According to Table 1, the dependence of the NLDN-reported peak current on distance in the present study is not negligible (determination coefficient = 0.50), if we take into account the whole dataset (see the last column in Table 1). This dependence, suggests a bias (characteristic of single-station, fixed-threshold-triggered field measurements in which the effective lower measurement limit in terms of source intensity increases with increasing distance from the measuring station) toward more intense sources being recorded from larger distances. However, if we analyze individual distance ranges (as done in Results section), such as 0–20 km and 100–150 km, we will find almost no distance dependence (determination coefficient = 0.02). In those distance ranges, we have recorded CIDs with both low and high NLDN-reported peak currents. For the 200–500 km distance range, the determination coefficient is higher than for the other ranges, but still relatively low (0.17).

**Table 1:** Number of NLDN-located compact intracloud discharges in different horizontal distance ranges, corresponding GM NLDN-reported peak currents, and determination coefficients between distance and peak current for each range of distances.

Distance range (km)	0–20	20–50	50–100	100–150	150–200	200–500	0–500
Number of CIDs	25	218	297	248	163	145	1096
NLDN-reported peak currents (GM), kA	20	21	24	36	54	67	33
Determination coefficient	2%	3%	5%	2%	7%	17%	50%

Determination coefficient is the square of correlation coefficient expressed in percent.

**Figure 1:**
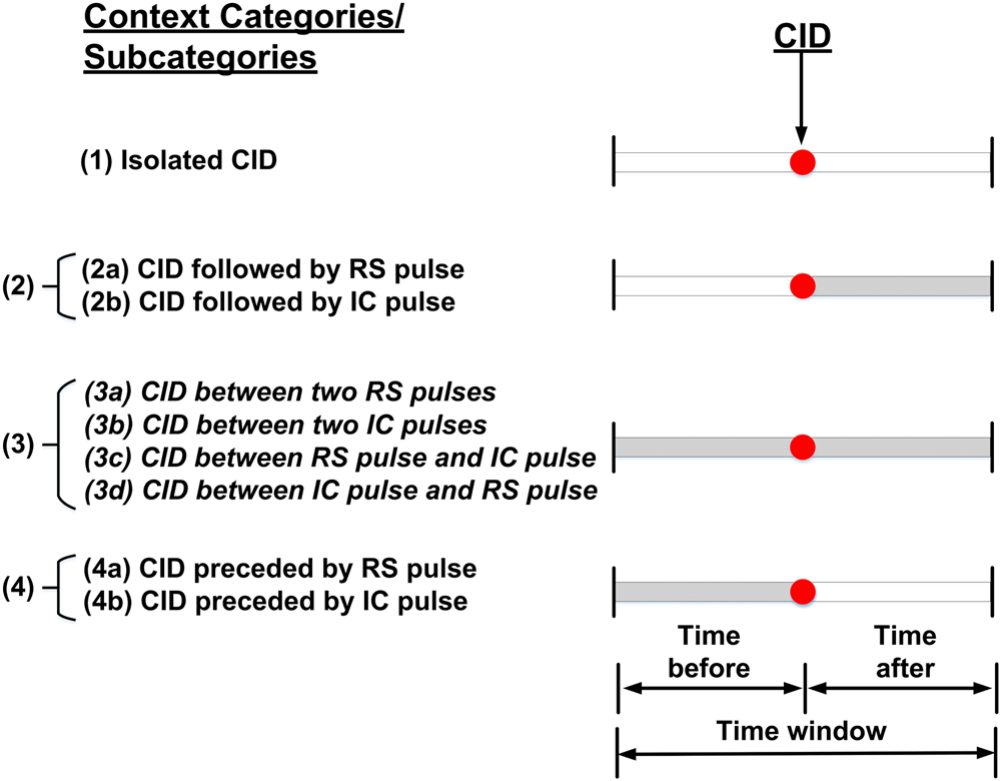
Illustration of different CID occurrence contexts. The red circle indicates the position of CID at the center of the time window and the shading indicates the occurrence of other lightning events (not CIDs) in different parts of the time window.

We consider four categories of CID context (see Fig. 1). Category 1 includes CIDs totally isolated in the specified time window. In Category 2, CIDs precede RS or/and IC pulses. In Category 3, CIDs both follow and precede RS or/and IC pulses. Finally, in Category 4, CIDs follow RS or/and IC pulses. For each category, four time windows and two search radii were considered.

**Figure 2:**
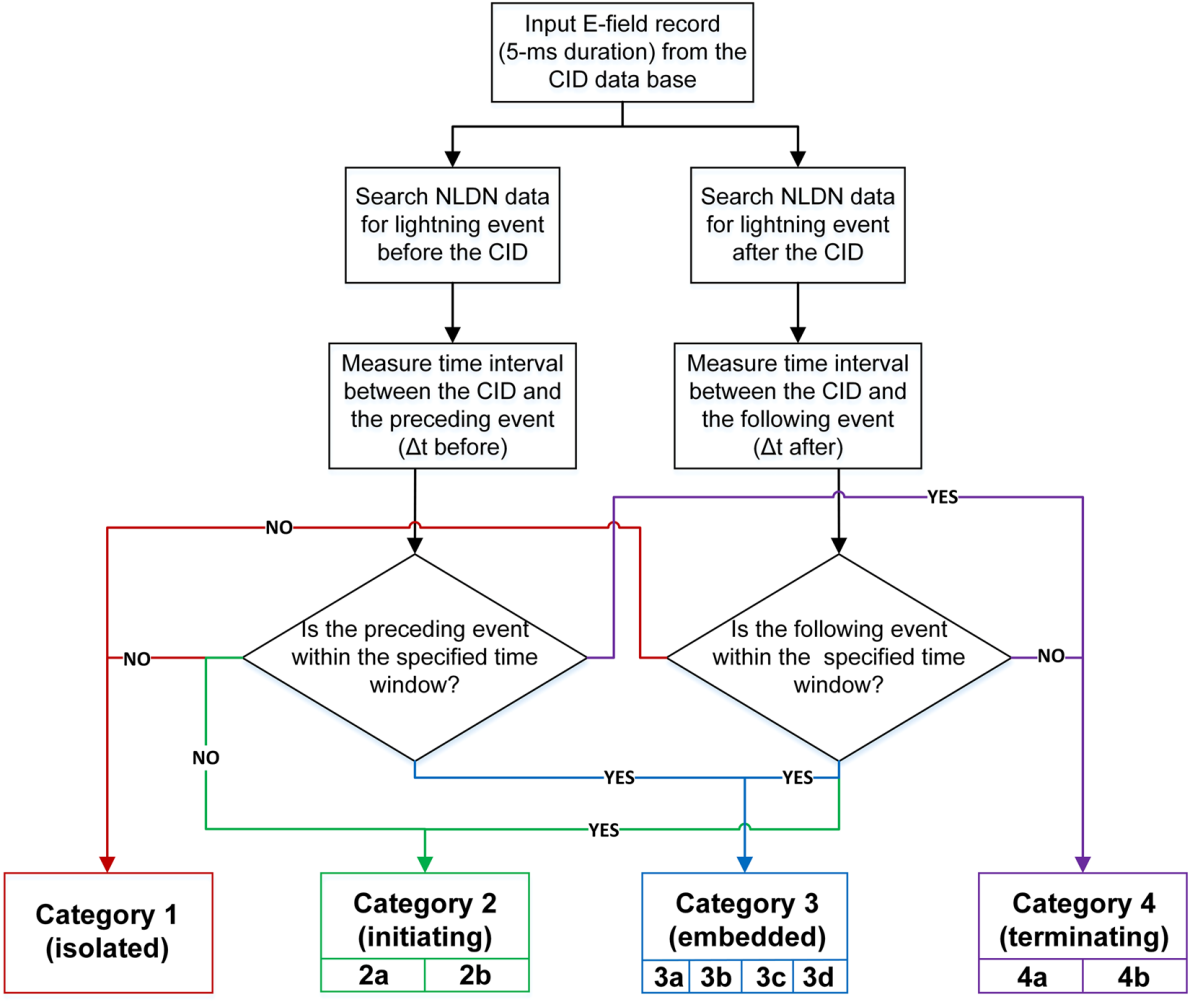
Flow-chart illustrating the automated algorithm used to classify CIDs into the four categories (and eight subcategories) illustrated in Fig. 1. This flow-chart was used for each search radius.

The flow-chart of the automated procedure used to classify CIDs into the four categories (and eight subcategories) depending on their occurrence relative to other forms of lightning activity is shown in Fig. 2. This procedure was originally designed for searching for the first (nearest in time to the CID) normal lightning event (pulse) either before or after the CID, but was later extended/modified for examination of complete flashes preceding or following the CID, as well as for identification of CID-precursors which precede normal lightning events at the same location by time intervals measured in seconds. In the analyses of complete flashes, the maximum number of pulses after the CID was 13, and before the CID it was 10, for the time window of ±500 ms and 10 km search radius.

For some of our CIDs, we could estimate heights using the characteristic pairs of ionospheric reflections, as done, for example, by Leal *et al*.^37^.

## Results

### Context of CIDs transporting negative charge upward (lower-level CIDs)

Results of classification of CIDs into nine categories/subcategories depending on their occurrence context are summarized in Table 2. Four different time windows and two search radii were used, which should cover essentially all the expected combinations of the temporal and spatial isolation needed for identification of the nine scenarios considered in this paper. One scenario that is outside the scope of this study is the occurrence of CID in groups of two or more within tens to hundreds of milliseconds. Nag *et al*.^6^ reported their CID pairs to occur with spatial separation greater than 10 km.

**Table 2:** Occurrence (in %) of 12 different CID contexts (4 categories and 8 subcategories) for 4 different time windows and 2 search radii.

Time window	±1000 ms		±500 ms		±100 ms		±10 ms	
Search radius	10 km	5 km	10 km	5 km	10 km	5 km	10 km	5 km
**Category 1 (isolated)**	**31%**	**44%**	**45%**	**56%**	**71%**	**78%**	**90%**	**92%**
**Category 2 (initiating)**	**43%**	**41%**	**37%**	**34%**	**21%**	**18%**	**8%**	**7%**
Subcategory 2a	6%	6%	5%	3%	1%	0.9%	1%	0.5%
Subcategory 2b	37%	36%	33%	30%	19%	17%	7%	6%
**Category 3 (embedded)**	**17%**	**7%**	**10%**	**5%**	**4%**	**2%**	**0**.**5%**	**0**.**5%**
Subcategory 3a	1%	0.4%	0.9%	0.3%	0.5%	0.2%	0.0%	0.0%
Subcategory 3b	11%	5%	7%	4%	3%	2%	0.4%	0.4%
Subcategory 3c	2%	1%	1%	0.7%	0.6%	0.5%	0.2%	0.2%
Subcategory 3d	2%	0.3%	0.5%	0.1%	0.3%	0.1%	0.0%	0.0%
**Category 4 (terminating)**	**9%**	**7%**	**8%**	**5%**	**5%**	**2%**	**2%**	**1%**
Subcategory 4a	2%	2%	2%	1%	1%	0.5%	0.7%	0.4%
Subcategory 4b	7%	5%	6%	4%	3%	2%	1%	0.7%
**All non-isolated**	**69%**	**56%**	**55%**	**44%**	**29%**	**22%**	**10%**	**8%**

Sample size = 1096.

As expected, the percentage of isolated CIDs (Category 1) is larger for the shorter search radii and increases with decreasing the time window. The percentage of isolated CIDs decreases from 92% for 5 km and ±10 ms to 31% for 10 km and ±1000 ms, this decrease being accompanied by an increase of the percentage of CIDs preceding (initiating) normal lightning events from 6.8% to 43%. Other CID occurrence contexts are relatively rare. If there is a preceding or/and following pulse, it is more likely to be an IC pulse than an RS pulse. In category 2b (see Fig. 1), CIDs precede IC pulses. This scenario may be indicative of CID initiating either an IC flash or CG flash, because IC pulses (associated with preliminary breakdown process) occur at the beginning of CG flashes. CIDs occurring between IC and RS pulses (Subcategory 3d), and CIDs occurring between RS pulses (Subcategory 3a) are the least likely scenarios.

The choice of search radius (5 or 10 km) seems to have relatively little effect on the results, particularly for shorter time windows. Clearly, a decrease in search radius leads to a larger percentage of isolated CIDs.

In the following, we present more detailed information on CID context for one specific time window, ±500 ms, and search radius of 10 km, which are similar to those used by Karunarathne *et al*.^7^ and appear to be optimal for studying association of CIDs with other forms of lightning activity. Tables 3 and 4 summarize information on the number of events (pulses before and after the CID) in each category, the geometric and arithmetic means of NLDN-reported peak currents for CIDs, the geometric and arithmetic means of the NLDN-reported peak current for events preceding and following the CIDs, the number of positive and negative IC and RS pulses that occurred before and after the CID, the arithmetic mean of the time interval between the CID and events occurring before and after it, and the arithmetic mean of the distance between the CID and events occurring before and after it.

**Table 3:** Characterization of events preceding CIDs (Categories 3 and 4) for ±500 ms time window and 10 km search radius.

	Sample size	CID		Preceding pulse													
		GM Ip (kA)	AM Ip (kA)	IC						RS						AM Δr (km)	AM Δt (ms)
				Max Ip	Min Ip	GM Ip	AM Ip	N_pos_	N_neg_	Max Ip	Min Ip	GM Ip	AM Ip	N_pos_	N_neg_		
Category 3 (embedded)	112	28	35	20	1.6	6.5	7.5	82	4	88	3.6	23	30	15	11	4.4	137
Category 4 (terminating)	86	33	43	28	0.9	6.4	7.7	62	5	62	1.5	15	23	12	7	5.1	204
All preceding	198	30	38	28	0.9	6.5	7.6	144	9	88	1.5	19	27	27	18	4.8	166

**Table 4:** Characterization of events following CIDs (Categories 2 and 3) for ±500 ms time window and 10 km search radius.

	Sample size	CID		Following pulse													
		GM Ip (kA)	AM Ip (kA)	IC						RS						AM Δr (km)	AM Δt (ms)
				Max Ip	Min Ip	GM Ip	AM Ip	N_pos_	N_neg_	Max Ip	Min Ip	GM Ip	AM Ip	N_pos_	N_neg_		
Category 2 (initiating)	409	34	39	30	1	6.0	6.8	348	11	78	1.4	23	29	28	22	3.0	159
Category 3 (embedded)	112	28	35	20	1.2	6.1	6.9	90	6	130	5.1	22	29	7	9	3.7	144
All following	521	33	38	30	1	6.0	6.8	438	17	130	1.4	22	29	35	31	3.2	155

In Tables 3 and 4, N_pos_ and N_neg_ denote the number of pulses reported by the NLDN as positive and negative, respectively. In the case of RS, the NLDN-reported polarity corresponds to the polarity of charge transported to ground, and in the case of IC pulses “positive” corresponds to transfer of negative charge upward (+IC) and “negative” to transfer of negative charge downward (−IC). Note that the NLDN polarity convention for IC pulses is such that our lower-level CIDs correspond to +ICs in NLDN reports and vice versa.

The largest geometric mean peak current corresponds to CIDs that appeared to initiate other lightning events (Category 2). The second largest geometric mean peak current corresponds to isolated CIDs (Category 1). NLDN-reported peak currents for CIDs tend to be three times larger than the peak currents of other events that occurred before or after them. If we consider only those “other” events classified by the NLDN as IC pulses, the NLDN-reported peak current (GM value) for CIDs is about five times larger than that for preceding and/or following events, and for other (preceding or following) events classified as RS pulses the CID peak current is about 1.5 larger. This trend makes CIDs unlikely to be missed and argues against the notion that CIDs initiate many or all ordinary lightning flashes (Rison *et al*.^21^), but could be difficult to detect. Note that the NLDN DE for CIDs was estimated^6^ to be 96%.

In category 3, where CIDs occur between two other events, those events were mostly IC pulses of the same polarity as that of the CID. It appears that CIDs are rarely related to CG flashes, with most of them being related to IC pulses. This finding is similar to that reported by Wu *et al*.^29^.

**Figure 3:**
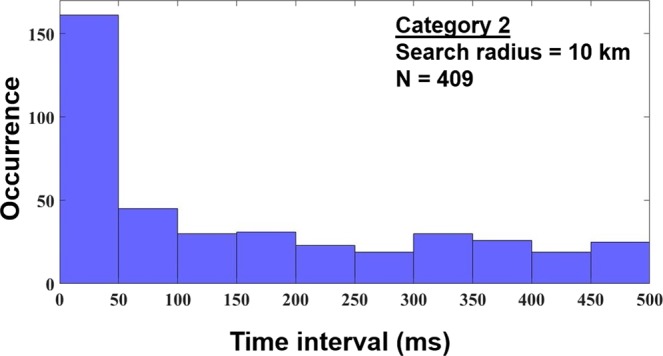
Histogram of time intervals (up to 500 ms) between the CID and the first pulse of the following lightning event (Category 2). Median = 97 ms.

**Figure 4:**
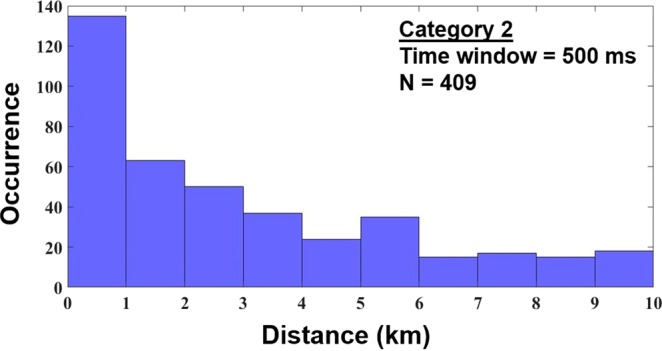
Histogram of distances (up to 10 km) between the CID and the source of the first pulse of the following lightning event (Category 2). Median = 2 km.

In Category 2, where CIDs precede (initiate) other lightning events, those events are usually (85%) IC pulses of the same polarity as that of the CID or, more rarely, positive RS pulses (7%). Most of these events (their first detectable pulse after the CID) occurred less than 50 ms after the CID (see Fig. 3) and within a horizontal distance less than 1 km from it (see Fig. 4). The median time interval and the median distance are 97 ms and 2 km, respectively.

**Figure 5:**
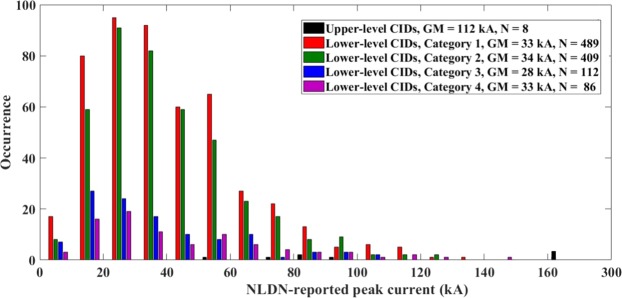
NLDN-reported peak currents for 4 categories of lower-level CID context and for all categories of upper-level CID context combined, shown in 10-kA bins. GM is the geometric mean peak current reported by the NLDN and N is the sample size. There are 3 upper-level CIDs between 160 and 300 kA.

Table 5 gives the lower-level CID occurrence contexts for different NLDN-reported peak current ranges. NLDN-reported peak currents for the 4 categories of lower-level CID context (also for all categories of upper-level CID context) are shown in 10-kA bins in Fig. 5. Geometric mean (GM) peak currents for each CID category are also presented in that Figure.

**Table 5:** Occurrence context of lower-level CIDs based on the ±500 ms time window and 10 km search radius for different lower-level CID peak current ranges.

	NLDN-reported peak current range (kA)						
	5–20	20–40	40–60	60–80	80–100	100–142	5–142
Category 1 (isolated)	45%	43%	47%	45%	38%	50%	45%
Category 2 (initiating)	31%	40%	40%	36%	36%	23%	37%
Category 3 (embedded)	16%	10%	7%	10%	13%	8%	10%
Category 4 (terminating)	9%	7%	6%	9%	13%	19%	8%
All non-isolated	55%	57%	53%	55%	62%	50%	55%
Sample Size	217	431	265	110	47	26	1096

As seen in Table 5, for any range of peak current, most of the lower-level CIDs occurred in isolation. For higher peak currents (100–142 kA), 50% of lower-level CIDs belong to Category 1, although the sample size (N = 26) is relatively small. The overall range of NLDN-reported peak currents for lower-level CIDs was 5 to 142 kA with geometric mean (GM) of 33 kA and arithmetic mean (AM) of 39 kA. Similar CID currents were reported by Karunarathne *et al*.^7^, who found a range of 2 to 126 kA with an AM of 32 kA. It is important to note that NLDN-reported currents for CIDs are estimated using an equation developed for RSs and, therefore, cannot be viewed as accurate. Possible errors involved are discussed by Nag *et al*.^38^. It is probably best to consider the CID current reported by the NLDN as a measure of source intensity expressed in kA, which is useful only for intercomparison of different events.

**Figure 6:**
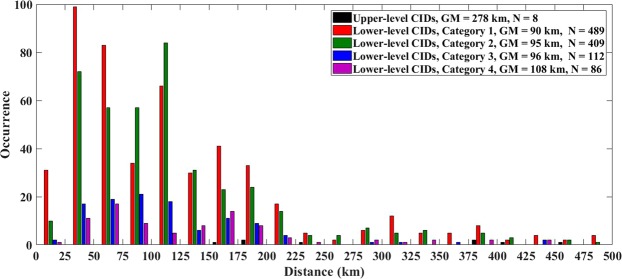
Distances at which CIDs were recorded for 4 categories of lower-level CID context and for all categories of upper-level CID context combined, shown in 25-km bins. GM is the geometric mean distance and N is the sample size.

Table 6 gives the occurrence context of lower-level CIDs in different distance ranges. The minimum and maximum distances were 9.3 and 495 km, respectively. Except for the range from 100 to 150 km, in all the ranges the greatest percentage of the lower-level CIDs occurred in isolation. In the 100–150 km range, the greatest percentage (46%) of lower-level CIDs preceded (initiated) normal lightning activity and only 39% were isolated. It is not clear what is special about the 100–150 km distance range. Figure 6 shows distances at which CIDs were recorded for the 4 categories of lower-level CID context (also for all categories of upper-level CID context combined) in 25-km bins.

**Table 6:** Occurrence context of lower-level CIDs based on the ±500 ms time window and 10 km search radius for different distance ranges.

	Distance range (km)						
	0-20	20–50	50–100	100–150	150–200	200–500	0–500
Category 1 (isolated)	84%	50%	39%	39%	45%	50%	45%
Category 2 (initiating)	84%	50%	39%	39%	45%	50%	45%
Category 3 (embedded)	0%	9%	13%	10%	12%	6%	10%
Category 4 (terminating)	4%	5%	9%	5%	13%	9%	8%
All non-isolated	16%	50%	61%	61%	55%	50%	55%
Sample Size	25	218	297	248	163	145	1096

We have additionally stratified our data set according to the 11 types of CID field waveforms (see Table A1) identified by Leal *et al*.^8^. The ±500 ms time window and 10 km search radius were used. The results are presented in Table A2. Lower-level CIDs whose electric field waveforms exhibit periodic variations (ringing) in the opposite polarity overshoot are mostly isolated, while those without ringing in the electric field waveforms tend to initiate normal lightning events.

We now discuss the CID occurrence context considering all the NLDN-detected pulses before and after the CID within the specified time window (not only the pulse nearest in time to the CID). Tables 7 and 8 summarize composition of pulse sequences before and after the CID for different occurrence contexts, based on the ±500 ms time window and 10 km search radius. Four scenarios are represented in Tables 7 and 8, “Only IC pulses”, “Only RS pulses”, “Both IC and RS pulses (IC pulse first)” and “Both IC and RS pulses (RS pulse first)”, for each of the four categories of CID occurrence context.

**Table 7:** Composition of pulse sequences during 500 ms before CID for categories 3 and 4 and 10 km search radius.

	Total number of CIDs	Only IC pulses				Only RS pulses				Both IC and RS pulses (IC pulse first)				Both IC and RS pulses (RS pulse first)			
		N	Min	Max	AM	N	Min	Max	AM	N	Min	Max	AM	N	Min	Max	AM
Category 3 (embedded)	112	81	1	6	1.8	23	1	8	1.4	5	2	10	4.0	3	2	9	5
Category 4 (terminating)	86	64	1	5	1.4	18	1	4	1.4	3	2	3	2.3	1	2	2	2
All before	198	145	1	6	1.6	41	1	8	1.4	8	2	10	3.4	4	2	9	4.3

**Table 8:** Composition of pulse sequences during 500 ms after CID for categories 2 and 3 and 10 km search radius.

	Total number of CIDs	Only IC pulses				Only RS pulses				Both IC and RS pulses (IC pulse first)				Both IC and RS pulses (RS pulse first)			
		N	Min	Max	AM	N	Min	Max	AM	N	Min	Max	AM	N	Min	Max	AM
Category 2 (initiating)	409	320	1	8	2.1	33	1	9	1.515	39	2	13	5.0	17	2	7	3.6
Category 3 (embedded)	112	82	1	7	2.0	9	1	7	2.111	14	2	11	4.4	7	2	12	5.4
All after	521	402	1	8	2.1	42	1	9	1.6	53	2	13	4.8	24	2	12	4.2

**Figure 7:**
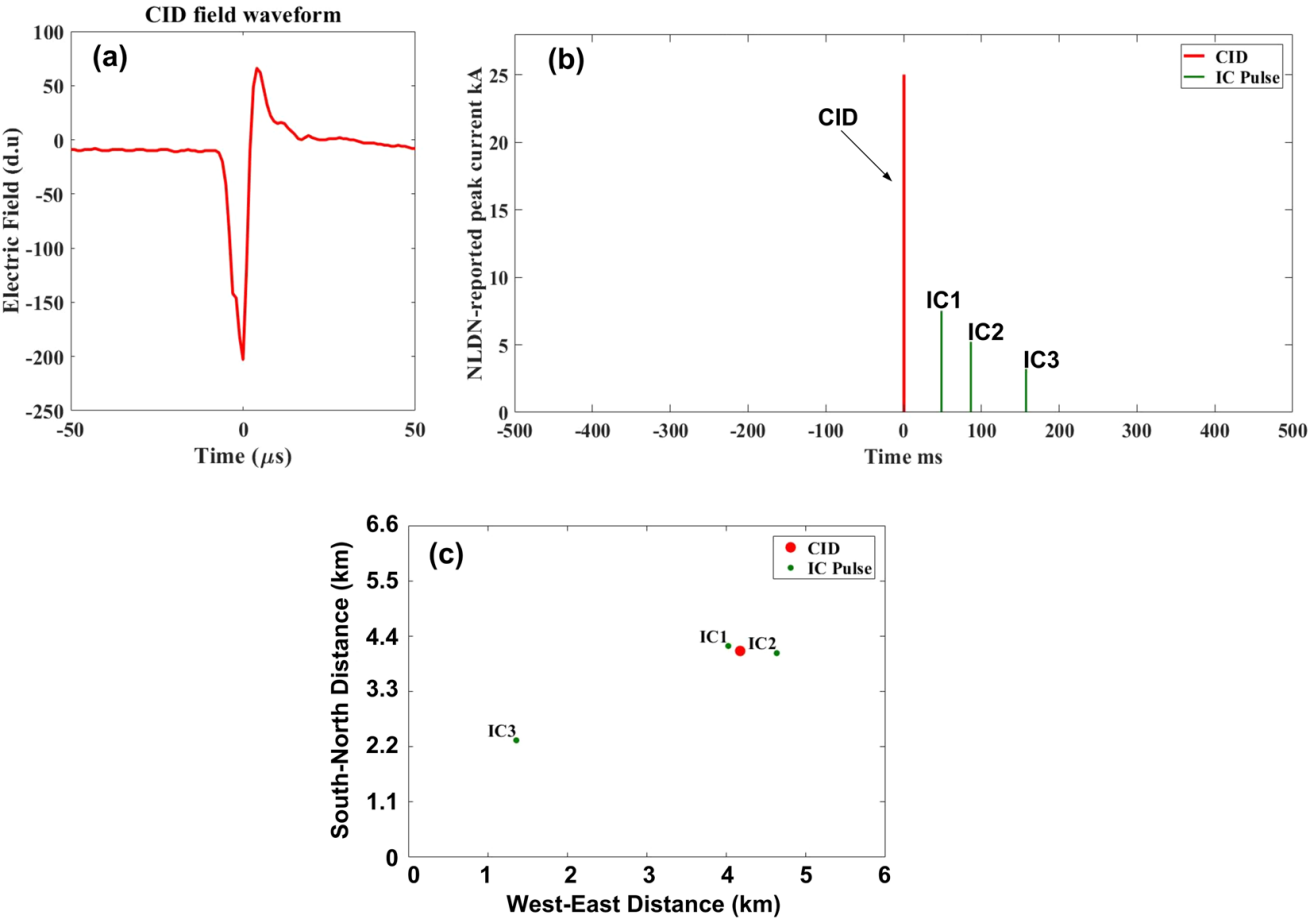
An example of lower-level CID that was followed by three IC pulses within 10-km search radius and ±500-ms time window. The CID apparently initiated an IC flash. (**a**) CID electric field waveform, (**b**) NLDN-reported peak currents for the CID and the following IC pulses, (**c**) NLDN-reported locations of the CID and the following IC pulses.

**Figure 8:**
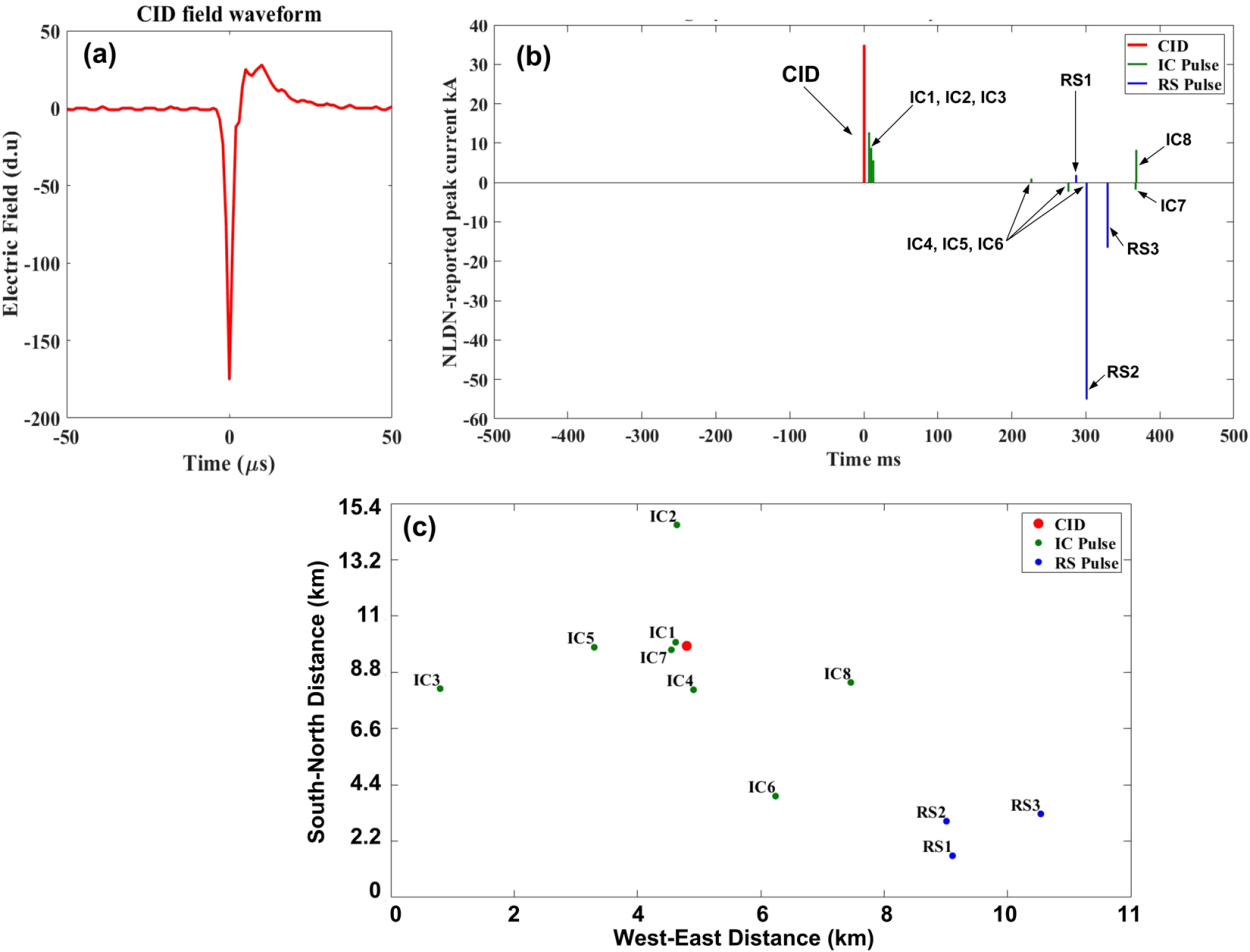
An example of lower-level CID that was followed by eight IC pulses and three RS pulses within 10-km search radius and ±500-ms time window. The CID apparently initiated a −CG flash. (**a**) CID electric field waveform, (**b**) NLDN-reported peak currents for the CID and the following IC and RS pulses, (**c**) NLDN-reported locations of the CID and the following IC and RS pulses. Note that RS1 transported positive charge to ground, while larger RS2 and RS3 were negative. It is possible that the small RS1 was actually a cloud pulse misclassified by the NLDN. This flash was apparently a hybrid one, initiated by the CID with IC1 through IC3 being the initial breakdown pulses of the IC part of the flash, which was followed by the CG part. Note that RS1 occurred 287 ms after the CID.

**Figure 9:**
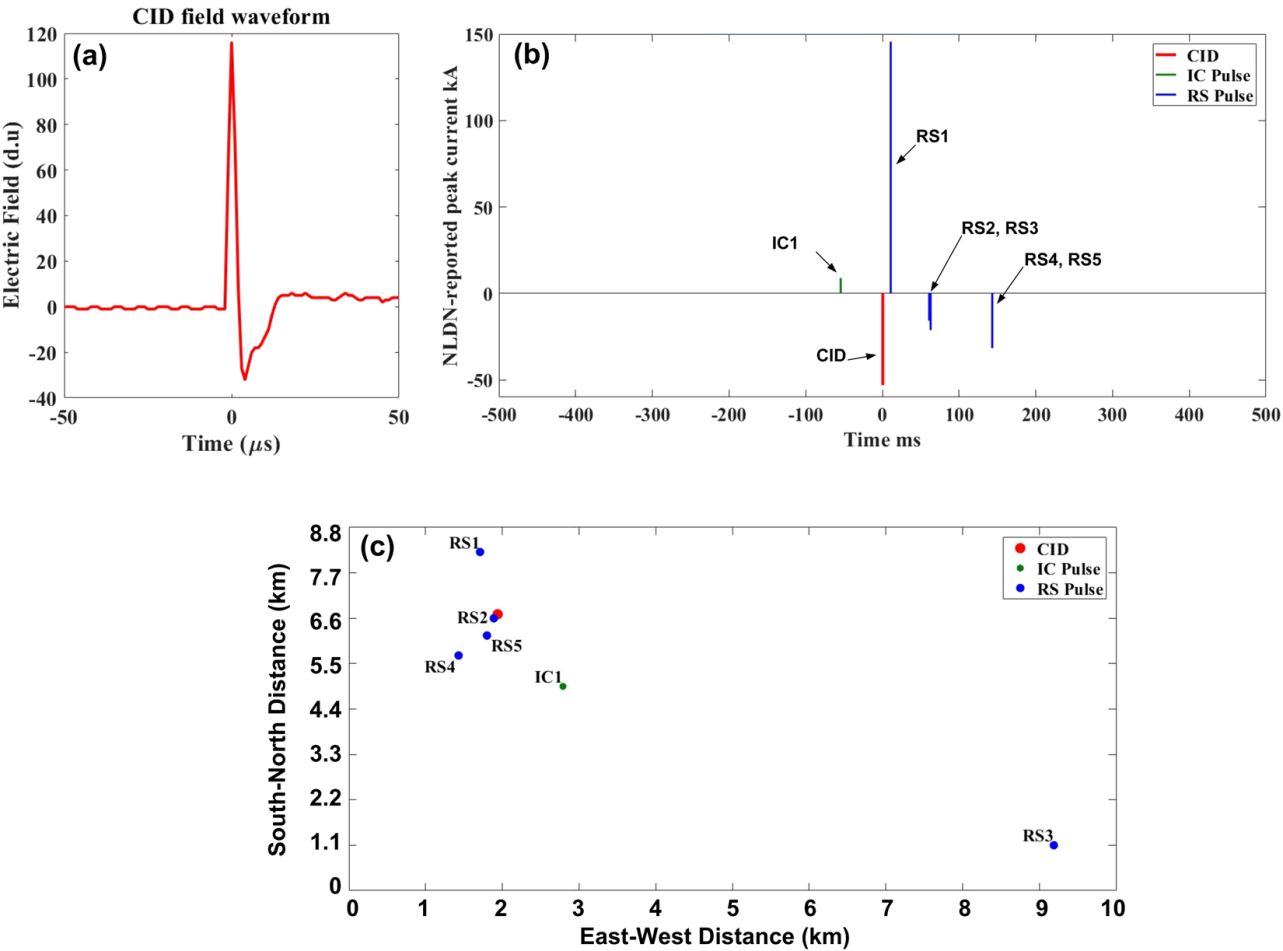
An example of upper-level CID that was preceded by one IC pulse and followed by five RS pulses for 10-km search radius and ±500-ms time window. (**a**) CID electric field waveform, (**b**) NLDN-reported peak currents for the CID and the preceding IC pulse and following RS pulses, (**c**) NLDN-reported locations of the CID and the preceding IC pulse and following RS pulses. Note that RS1 transported positive charge to ground, while the other 4 strokes were negative. The CID occurred 11 ms prior to RS1 at (above) the position of RS2, which was 1.6 km away from that of RS1. RS3 occurred 8.9 km away from RS2, while RS4 and RS5 were close (within 1 km) of RS2.

According to Tables 7 and 8, when CIDs appear to initiate other lightning events (Category 2), those events are mainly IC flashes (320 out of 409 or 78%) with a few (Mean = 2.1) detectable IC pulses. An example of such scenario is shown in Fig. 7. Another scenario (39 out of 409 or 10%) in Category 2 is when the CID is followed by IC pulses, but lightning develops into a CG flash with one or more RSs. In this scenario, the total (IC + RS) number of pulses is higher (Mean = 5). This scenario is most likely the case of CID initiating a CG flash producing a PB pulse train followed by RS pulses (see an example shown in Fig. 8). Our CIDs in Category 3 most of the time (81 out of 112 or 73%) occurred in the middle of an IC flash, and in Category 4 they usually terminated an IC flash (64 out of 86 or 74%). Note that in Figs 7 and 8 (also in Fig. 9), the electric field waveforms of CIDs are shown using the atmospheric electricity sign convention (negative polarity corresponds to negative charges moving upward and positive polarity to positive charges moving upward), while the polarity of current is shown as it was reported by the NLDN.

### Can isolated lower-level CIDs actually be precursors

Rison *et al*.^21^ observed CID-like events (although relatively small) that were followed some seconds later by normal lightning (usually IC flash) at the same location. They referred to those events as precursors (PCs). Such events could also occur in isolation, being apparently indicative of attempted breakdown that did not develop into a full-fledged lightning flash. According to Rison *et al*.^21^, the higher-intensity isolated CIDs are just the higher-power tail of the spectrum of lower-intensity precursor events.

In the following, we checked if any of our isolated CIDs for the time window of ±1000 ms and 10 km search radius (341 events) can be classified as precursors; that is, if they were followed by normal lightning events at essentially the same location after a time interval measured in seconds. An isolated (within ±1000 ms) CID was classified as precursor if it was followed by normal lightning events at essentially the same location (within 0.5 km radius) after a time interval >1 s. Specifically, we employed 3 time intervals after the CID, 5, 10, and 30 s. Compared to our analyses of CID occurrence context in Results section, in this section we tightened the distance criterion and loosened (in the forward direction) the time criterion. The results are summarized in Table 9, with data for the 1-s time interval being included as reference.

**Table 9:** Isolated CIDs (1-s interval) vs. CID-precursors (5 and 10-s intervals).

Time interval	1 s			5 s			10 s		
	N	Percentage	GM peak current, kA	N	Percentage	GM peak current, kA	N	Percentage	GM peak current, kA
Isolated CIDs	341	100%	31	336	99%	31	329	96%	31
CID-precursors	0	0%	—	5	1%	50	12	4%	45
All data combined	341	100%	31	341	100%	31	341	100%	31

We did not consider search radii smaller than 0.5 km, because the median location error of the NLDN is of the order of a few hundred meters for CG strokes^36,39^ and probably larger for IC pulses. It is worth noting that the 0.5 km radius is two-dimensional and that normal lightning events likely occurred at heights that are appreciably lower than the CID-precursor heights.

As seen in Table 9, the majority of CIDs that had been found in Section 3.1 to be isolated within 10 km and ±1000 ms remained isolated when the search radius was decreased to 0.5 km and the time interval after the CID was increased to 5 or 10 s. The percentage of CID-precursors increased from 1% to 4%. For the 12 CID-precursors that were found for the time interval of 10 s, the median value of the time interval between the CID and the first pulse of the following lightning event was 6.7 s. It was found that the GM peak current reported by the NLDN for CID-precursors was higher than for isolated −CIDs.

### CIDs transporting positive charge upward (upper-level CIDs)

The analyses presented in the previous two subsections were performed for CIDs that were associated with negative charge moving upward (lower-level CIDs). Here we examine CIDs transporting positive charge upward (upper-level CIDs). In our data set, there are only 8 upper-level CIDs, which corresponds to only 0.7% of the total number (1104) of CIDs, which we recorded at LOG in 2016 and for which NLDN data were available.

Upper-level CIDs were found (as expected) to occur at higher altitudes than lower-level ones^13,14,40,41^. According to Smith *et al*.^40^, lower-level CIDs occur between 7 and 15 km above the ground level and upper-level CIDs between 15 and 20 km. Most of upper-level CIDs studied by Wu *et al*.^13^ occurred at heights ranging from 16 to 19 km vs. 8 to 16 km for most of lower-level CIDs. A number of studies have shown that upper-level CIDs occur less frequently than lower-level CIDs. Jacobson and Heavner^42^ reported that 23% of 103,240 CIDs recorded in Florida during 1999–2002 were upper-level CIDs. Wiens *et al*.^43^ reported the same percentage (23%) of upper-level CIDs recorded by LASA (Los Alamos Sferic Array) during May to July in 2005 in the U.S. Great Plains. Wu *et al*.^17^ reported a total of 254 CIDs in Japan in the summer of 2012, of which only 9% were upper-level CIDs. Lü *et al*.^16^ reported that only 493 CIDs were recorded during the 2 year observation period in the northernmost region (51°N) of China, and no upper-level CIDs were observed; they stated that upper-level CIDs are rare in higher-latitude regions. Wu *et al*.^13^ reported that upper-level CIDs produced, on average, larger electric field changes than lower-level CIDs and, by inference, higher peak currents. As noted above, currents reported by the NLDN for CIDs should be viewed with caution.

Upper-level CID occurrence contexts, as well as NLDN-reported peak currents and heights estimated using the pairs of ionospheric reflections are summarized in Table 10. Out of 8 upper-level CIDs, 4 (50%) were isolated, 2 (25%) were initiating, and 2 (25%) were embedded into normal lightning activity. No upper-level CIDs were preceded by normal lightning events.

**Table 10:** Characterization of upper-level CIDs based on the ±500-ms time window and 10-km search radius.

	NLDN-reported peak current, kA (absolute value)					Height, km				
	Sample size	Min	Max	GM	AM	Sample size	Min	Max	GM	AM
Category 1 (isolated)	4	78	210	106	116	2	17	17	17	17
Category 2 (initiating)	2	166	290	219	228	2	16	17	17	17
Category 3 (embedded)	2	53	80	65	67	1	18	18	18	18
Category 4 (terminating)	0	—	—	—	—	0	—	—	—	—
All data combined	8	53	290	113	132	5	16	18	17	17

For all data combined, the standard errors in AM values of peak current (N = 8) and height (N = 5) are 22% and 2.1% of the AM value, respectively.

According to Table 10, upper-level CIDs indeed have considerably higher NLDN-reported peak currents than lower-level CIDs (see also Fig. 5). Only 5 upper-level CIDs had detectable pairs of ionospheric reflection that could be used for estimating source heights, which ranged from 16 to 18 km. The horizontal distances for these 5 upper-level CIDs ranged from 182 to 465 km. For the two upper-level CIDs in Category 2, one was followed by one IC pulse and the other was followed by one RS pulse. The occurrence context of one of the upper-level CIDs from Category 3 is illustrated in Fig. 9.

### Heights at which CIDs occur

According to Wu *et al*.^29^, “lightning initiator CIDs” (our Category 2) tend to occur at lower altitudes than “normal” CIDs. In order to investigate this trend in more detail, we computed the heights of 209 CIDs that showed detectable pairs of skywaves in their electric field signatures. We computed the source height based on the methodology used by Leal *et al*.^37^ and Smith *et al*.^5^, among others. In that methodology, the Earth’s curvature is neglected which is justifiable for horizontal distances less than 200 km or so, but not necessary for larger distances. Because of that, we split the 209 events into two different distance ranges, 75 to 200 km and 200 to 495 km. Table 11 gives the statistics on lower-level CID heights for the four categories of lower-level CID occurrence context.

**Table 11:** Inferred source heights for different lower-level CID occurrence contexts based on the ±500 ms time window and 10 km search radius.

	Source height in km for horizontal distance range of 75–200 km						Source height in km for horizontal distance range of 200–495 km						Source height in km for horizontal distance range of 75–495 km					
	Sample size	Max	Min	AM	GM	SE	Sample size	Max	Min	AM	GM	SE	Sample size	Max	Min	AM	GM	SE
Category 1 (isolated)	50	15	11	13	13	0.1	48	14	10	12	12	0.2	98	15	10	13	13	0.1
Category 2 (initiating)	45	14	6	11	11	0.3	30	14	8	11	12	0.3	75	14	6	11	11	0.2
Category 3 (embedded)	13	15	11	13	13	0.3	5	14	12	13	13	0.2	18	15	11	13	13	0.3
Category 4 (terminating)	9	15	13	14	14	0.3	9	14	11	13	13	0.3	18	15	11	13	13	0.2
All non-isolated	67	15	6	12	12	0.2	44	14	8	12	12	0.2	111	15	6	12	12	0.2
All data combined	117	15	6	12	12	0.2	92	14	8	12	12	0.1	209	15	6	12	12	0.1

AM – Arithmetic mean; GM – Geometric mean; SE – Standard error (standard deviation divided by $\sqrt{N}$, where N is the sample size).

**Figure 10:**
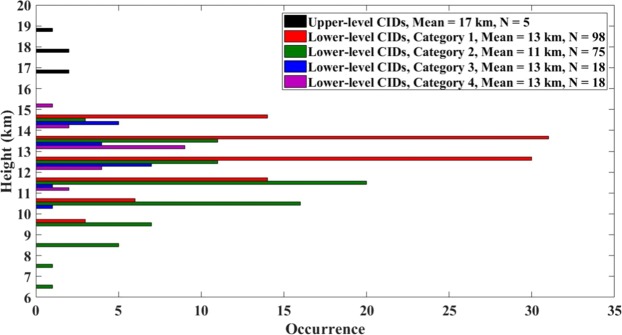
CID heights for 4 categories of lower-level CID occurrence context and for all categories of upper-level CID context combined, shown in 1-km bins. N is the sample sizes.

According to Table 11, for any range of distances, lower-level CIDs in Category 2 indeed occur at lower heights. For all data combined, the AM and GM heights of CIDs in Categories 1, 3 and 4 are the same and equal to 13 km, while for lower-level CIDs in Category 2 they are equal to 11 km. The 2-km difference is statistically significant, since the standard error (SE) is smaller than 0.4 km for all categories. Figure 10 shows heights for 4 categories of lower-level CID context and for all categories of upper-level CID context combined. Clearly, the heights of lower-level CIDs initiating normal lightning events (Mean = 11 km) tended to be lower than for all other CID categories, including isolated, embedded, and terminating events (Mean = 13 km for each of those 3 categories). Upper-level CIDs transporting positive charge upward occurred at heights ranging from 16 to 19 km, all being above the maximum height of lower-level CIDs transporting negative charge upward, whose range of heights was 6 to 16 km.

The mean height for our isolated lower-level CIDs observed in Florida was 13 km, which is not far from the typical height (11 km) for isolated lower-level CIDs observed in China by Wang *et al*.^44^ and similar to 13.4 km reported for Japanese storms by Wu *et al*.^29^. For the initiating lower-level CIDs, mean/typical heights in Florida, China, and Japan are 11 km, 11–12 km, and 7.9 km, respectively. Interestingly, about one-third of heights for the initiating lower-level CIDs in Japan were below 7 km vs. none in China and only 1 (1.3%) in Florida.

## Discussion

It is difficult to compare the CID context reported from different studies, because of the use of different criteria, particularly different time scales on which CIDs appear to occur in isolation. Nag *et al*.^6^, who used the time window with 100 ms prior and 400 ms after the CID, found that 73% of Florida CIDs occurred in complete isolation. Wu *et al*.^29^ investigated CIDs that belong to our Category 2. They termed these events Lightning-Initiator Narrow Bipolar Events (INBEs). They reported that out of 827 CIDs recorded in their campaign, 638 (77%) transported negative charge upward (same as the majority of CIDs considered here). Of the latter 638, 103 (12%) were INBEs and 538 (88%) were “normal” CIDs; that is, they did not initiate lightning flashes. It is not clear if those 535 were isolated or could be preceded by or embedded in other lightning activity. They did not specifically report the percentage of isolated CIDs. Wu *et al*.^29^ reported that their INBEs were always followed by IC pulses of the same polarity as that of INBE, starting within 10 ms of INBE and lasting for several to tens of milliseconds. The sources of IC pulses propagated predominantly upward over relatively short distances of the order of a few kilometers. The resultant lightning events were mostly IC flashes (93 out of 103 or 93%), with 2 flashes developing into +CGs and 5 into –CGs. Otherwise being similar, INBEs clearly tended to occur at lower altitudes compared to “normal” CIDs. Most of INBEs occurred bellow 10 km (Mean = 7.9 km), while most of “normal” CIDs occurred above 10 km (Mean = 13.4 km). However, this trend was not observed by Wang *et al*.^44^. Our results on isolated and initiating CID heights are consistent with those of Wu *et al*.^29^ and extend them to two additional categories, embedded and terminating CIDs.

The only study to date, in which multiple categories of CID context were considered and spatial constraints used (similar to our study) is the one performed by Karunarathne *et al*.^7^. Smith *et al*.^5^, who investigated CIDs in New Mexico, USA, using electric field records with duration of 4 to 10 ms (occasionally 20 or 50 ms) reported that most of their events occurred in isolation. A summary of the results of previous studies of CID context along with our results (for ±500-ms time window and 10-km search radius) is given in Table 12. Clearly, our results for 10-km search radius and ±500-ms time interval are consistent with those of Karunarathne *et al*.^7^.

**Table 12:** Summary of CID occurrence context found in different studies.

Reference	Location	Time window	Search radius	Sample Size	Category 1	Category 2	Category 3	Category 4
NAG *et al*.^23^	Florida, USA	−100 ms, +400 ms	—	157	73%	—	—	—
WANG *et al*.^44^	Hengdian, China			236	14%	55%	31%	<1%
WU *et al*.^29^	Osaka, Japan	±100 ms	—	638	—	12%	—	—
KARUNARATHNE *et al*.^7^	Florida, USA	±660 ms	10 km	226	37%	38%	15%	10%
Present study	Florida, USA	±500 ms	10 km	1096	45%	37%	10%	8%

Overall, our analysis indicates that CIDs tend to be solitary events and have wideband electromagnetic field signatures whose amplitudes are larger than those of normal lightning events. Further, they are relatively rare (about 3% of all lightning events recorded with our 5-ms time window field measuring system). These findings are not in support of actively debated hypothesis that many or possibly all lightning flashes are initiated by CIDs or CID-like processes.

It appears that CIDs can initiate (or lead to the initiation of) not only normal lightning, but also the so-called transient luminous events (TLEs) developing from clouds toward the ionosphere, which include blue starters, blue jets, and gigantic jets. Specifically, lower-level CIDs (occurring between the main negative and main positive charge regions) were reported to precede gigantic jets by some hundreds of milliseconds^45–47^. More recently, Chou *et al*.^48^ observed 6 “blue luminous events” that were accompanied by upper-level CIDs, occurring between the main positive and screening negative charge regions, at heights ranging from 16 to 18 km above ground level. Similarly, Liu *et al*.^49^ reported on 6 “blue discharges” each occurring within 1 ms (their time uncertainty) after an upper-level CID occurring at a height in the 15 to 18 km range above ground level. Interestingly, while upper-level CIDs appear to directly initiate upward-jet-type events, lower-level CIDs do so via normal ICs lasting some hundreds of milliseconds.

## Summary

1. The occurrence context of compact intracloud discharges (CIDs) was examined in detail, using electric field waveforms of CIDs and corresponding NLDN data. A total of 1096 CIDs transporting negative charge upward (lower-level CIDs) and 8 CIDs transporting positive charge upward (upper-level CIDs) recorded in Florida in 2016 were analyzed. The analyses were performed for 2 search radii, 5 and 10 km, and for 4 different time windows centered at the time of CID, ±1000, ±500, ±100, and ±10 ms.
2. The percentages of isolated lower-level CIDs decreased from 92% for 5 km and ±10 ms to 31% for 10 km and ±1000 ms, this decrease being accompanied by an increase of the percentage of CIDs preceding (initiating) normal lightning events from 6.8% to 43%. Other lower-level CID occurrence contexts were relatively rare. For 10-km search radius and ±500 ms time window, 46% of lower-level CIDs were isolated and 37% appeared to initiate normal lightning events. For the latter CID category, normal lightning events mostly occurred within 2-km radius and less than 100 ms after the CID.
3. NLDN-reported peak currents for isolated lower-level CIDs were similar to those initiating normal lightning events (GM peak current values were 33 and 34 kA, respectively). Lower-level CIDs that occurred in association with other lightning events (not in isolation) tended to have three times higher NLDN-reported peak currents than the IC or RS pulses following or preceding them.
4. It was found that about 2% (5%) of isolated CIDs could be viewed as precursors, because they apparently initiated normal lightning events at essentially the same location (within 500-m radius) when the search time interval after the CID was increased to 5 s (10 s). The GM peak current reported by the NLDN for CID-precursors was found to be higher than for isolated CIDs.
5. The heights of lower-level CIDs initiating normal lightning events (Mean = 11 km) tended to be lower than for all other CID categories, including isolated, embedded, and terminating events (Mean = 13 km for each of those 3 categories).
6. Embedded (8 lower-level and 1 upper-level) CIDs appeared to influence the course of the lightning flash, as first reported by Nag *et al*.^6^. An example is shown in Fig. 9.
7. Lower-level CIDs whose electric field waveforms exhibit periodic variations (ringing) on the opposite-polarity overshoot are mostly isolated, while those without ringing in the electric field waveforms tend to initiate normal lightning events.
8. Upper-level CIDs (a) occurred at heights ranging from 16 to 19 km, all being above the maximum height of lower-level CIDs, whose range of heights was 6 to 16 km, and (b) had considerably higher NLDN-reported peak currents: 113 kA vs. 33 kA (GM values).
9. Overall, our analysis indicates that CIDs tend to be solitary events and have wideband electromagnetic field signatures whose amplitudes are larger than those of normal lightning events. Further, they are relatively rare (about 3% of all lightning events recorded with our 5-ms time window field measuring system). These findings are not in support of the hypothesis that many or possibly all lightning flashes are initiated by CIDs or CID-like processes.

## Methods

In the LDWSS, electric fields were sensed with a 0.8-m long whip antenna followed by a pre-amplifier. The pre-amplifier included an integrator and a unity-gain, low-noise amplifier. The signal from the pre-amplifier was transmitted, via a 10-m long coaxial cable, to the main unit, which was installed inside the LOG cupola and consisted of analog signal conditioning circuitry and digitizing system. The conditioning circuitry included a 500-kHz low-pass anti-aliasing filter, a 30-Hz high-pass filter, and a programmable gain amplifier (PGA). The sampling rate of the digitizing system was 1 MHz. The digitizing system had an RTC (Real Time Clock) which was synchronized with a GPS module.

The U.S NLDN is a lightning detection network, presently operated by Vaisala, which has over 100 sensors installed in the contiguous USA. The sensors are connected to a central processor that provides the time, polarity, peak current, lightning type (IC pulse or return-stroke (RS) pulse in a CG flash) and location for each lightning event detected by the network. Recently Zhu *et al*.^36^, evaluated the performance characteristics of the NLDN in its current configuration (after the 2013 upgrade), based on optical and electric field data acquired at LOG. They found that the detection efficiency (DE) for 153 ground truth IC events was 33% (50/153). For isolated IC events (complete IC flashes), they found the DE to be as high as 73% (19/26). In Zhu *et al*.’s^36^ study, the “IC event” is defined as a sequence of IC pulses that may or may not be accompanied (preceded or followed) by CG strokes, and the “complete IC flash” is defined as an isolated IC event; that is, a complete sequence of IC pulses that is not accompanied by CG strokes. An “IC event”, as opposed to “complete IC flash”, can be part of CG flash. One example of such “IC event” is the preliminary breakdown pulse train, which is clearly an IC event occurring in a CG flash that is followed by one or more CG strokes. Zhu *et al*.^36^ did not consider the DE for individual IC pulses. They also estimated the classification accuracy (CA) for IC events in general and for complete IC flashes, which were 86% and 95%, respectively. For CG return strokes, the DE was found to be 92%, and the CA was also 92%.

## Supplementary information

Appendix
