# Supplementary material for: A study of the context in which compact intracloud discharges occur
Source: Sci Rep. 2019 Aug 21;9:12218. doi: 10.1038/s41598-019-48680-6 (PMC6704157; doi:10.1038/s41598-019-48680-6)
Supplement: Supplementary file 1 — Appendix [file 41598_2019_48680_MOESM1_ESM.pdf]

Appendix to "A study of the context in which compact intracloud discharges occur"

Adonis F. R. Leal[1*] and Vladimir A. Rakov[2,3]

[1]Department of Electrical and Biomedical Engineering, Federal University of Para (UFPA), Belém, Brazil, adonisleal@ufpa.br
[2]Department of Electrical and Computer Engineering, University of Florida (UF), Gainesville, USA, rakov@ufl.edu
[3]Moscow Institute of Electronics and Mathematics, National University Higher School of Economics, Moscow, Russia

Leal *et al.*[1] examined the occurrence of different types of electric field waveforms produced by lower-level CIDs in Florida. Both lower- and upper-level CIDs are illustrated in the context of typical cloud charge structure in Fig. A1. Leal *et al.*[1] have identified a total of 11 waveform types which are characterized and illustrated in Table A1. The occurrence context (Categories 1 through 4) of CIDs with electric field waveforms of 11 types is presented in Table A2. The ±500-ms time window and 10 km search radius were used. Examined here are only lower-level CIDs. Among the 8 upper-level CIDs, 7 exhibited smooth bipolar waveforms (Type 1) and 1 had a waveform with ringing on the opposite-polarity overshoot (Type 2).

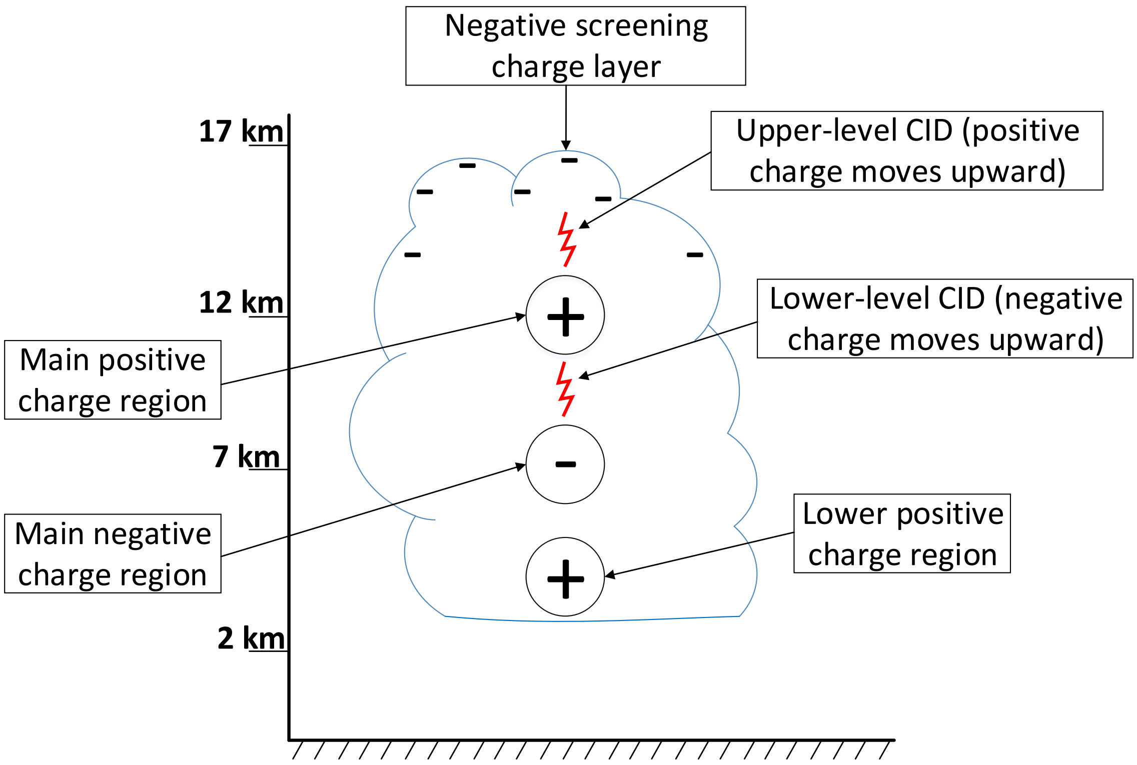

Fig. A1. Illustration of lower- and upper-level CIDs, producing, respectively, negative and positive initial half-cycles in their vertical electric field waveforms (based on the atmospheric electricity sign convention). Adapted from Leal *et al.*[1].

TABLE A1

Classification of wideband electric field waveforms produced by CIDs. Adapted from Leal *et al.*[1].

<table>
<tr><th rowspan="2">CID Type</th><th rowspan="2">Description</th><th rowspan="2">Representative Waveform</th><th colspan="2">Occurrence</th></tr>
<tr><th>Number</th><th>Percentage</th></tr>
<tr><td>1</td><td>Smooth bipolar waveform</td><td>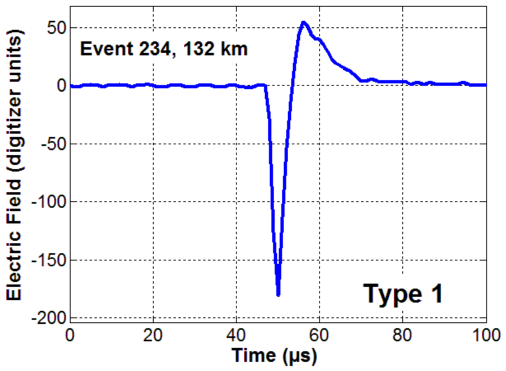
</td><td>285</td><td>26.00%</td></tr>
<tr><td>2</td><td>Smooth initial half-cycle, ringing on the opposite-polarity overshoot</td><td>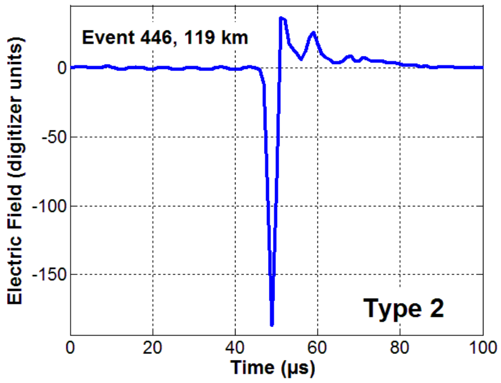
</td><td>463</td><td>42.24%</td></tr>
<tr><td>3</td><td>Smaller peak after the main one, smooth overshoot</td><td>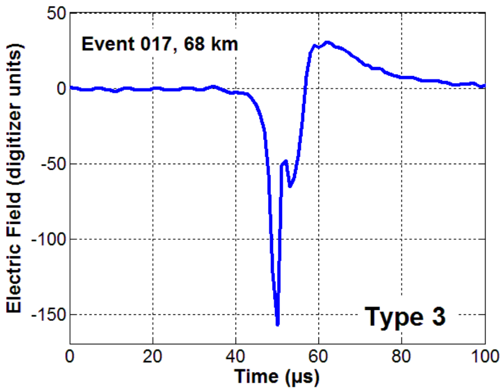
</td><td>96</td><td>8.76%</td></tr>
<tr><td>4</td><td>Smaller peak before the main one, smooth overshoot</td><td>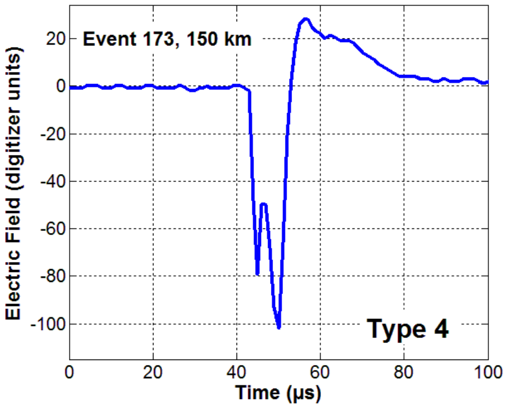
</td><td>73</td><td>6.66%</td></tr>
</table>

TABLE A1

Classification of wideband electric field waveforms produced by CIDs. Adapted from Leal *et al.*[1].

CID Type	Description	Representative Waveform	Occurrence	
			Number	Percentage
5	Same as 3, but with ringing on the overshoot	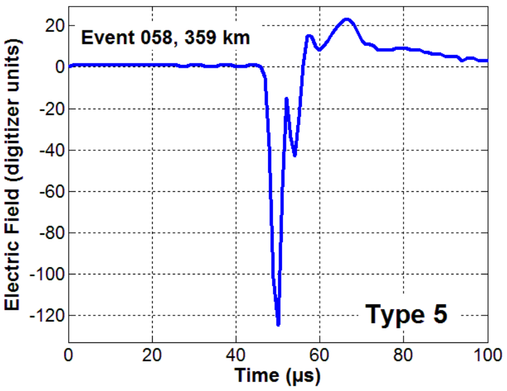	80	7.30%
6	Same as 4, but with ringing on the overshoot	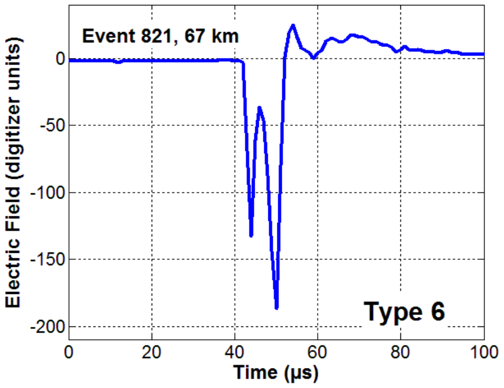	34	3.10%
7	Smooth bipolar waveform with slow front	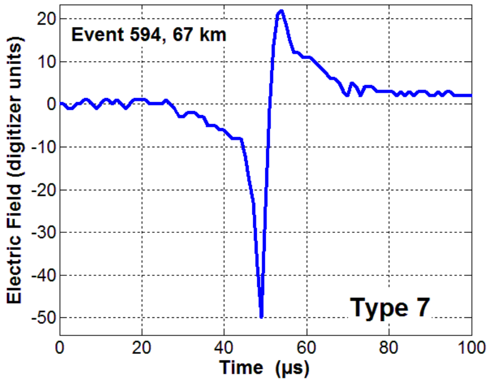	44	4.01%
8	Unipolar waveform	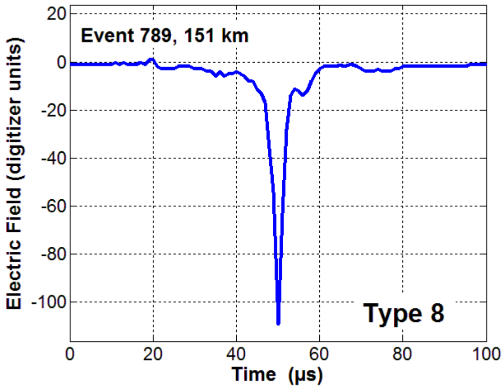	2	0.18%

TABLE A1

Classification of wideband electric field waveforms produced by CIDs. Adapted from Leal *et al.*[1].

CID Type	Description	Representative Waveform	Occurrence	
			Number	Percentage
9	Slow front, very large ringing on the overshoot	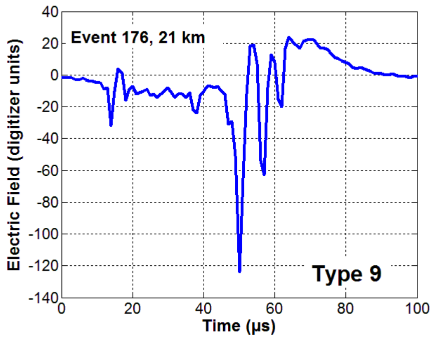	4	0.36%
10	Multi-peak initial half-cycle, smooth overshoot	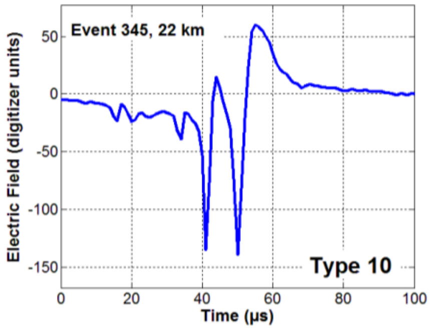	2	0.18%
11	Characteristic bipolar waveform preceded by a smaller-pulse train	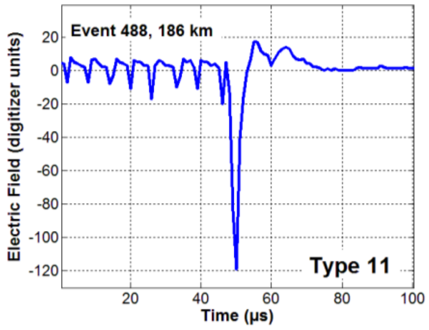	13	1.19%
**All 11 types**	**-**	**-**	**1096**	**100%**

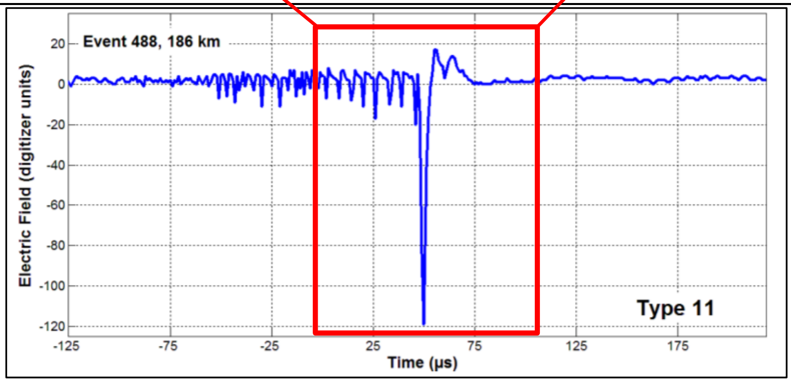

TABLE A2. Occurrence contexts (Categories 1 through 4) of CIDs for 11 types of CID field waveforms.

CID field waveform type	Sample size	Percentage	Percentage in different CID context categories			
			Category 1 (isolated)	Category 2 (initiating)	Category 3 (embedded)	Category 4 (terminating)
**1**	285	26.0%	37%	48%	9%	6%
**2**	463	42.2%	51%	32%	8%	9%
**3**	96	8.8%	36%	44%	15%	5%
**4**	73	6.7%	40%	45%	10%	5%
**5**	80	7.3%	61%	25%	4%	10%
**6**	34	3.1%	53%	38%	9%	0%
**7**	44	4.0%	18%	34%	36%	11%
**8**	2	0.2%	100%	0%	0%	0%
**9**	4	0.4%	50%	25%	0%	25%
**10**	2	0.2%	50%	50%	0%	0%
**11**	13	1.2%	15%	0%	54%	31%
**1-11**	**1096**	**100%**	**489 (45%)**	**409 (37%)**	**112 (10%)**	**86 (8%)**

According to Table A2, most of the CIDs with field waveforms of types 2, 5, and 6, which are characterized by periodic variations (ringing) on the overshoot, were isolated (Category 1). Most of the CIDs with field waveforms without ringing (types 1, 3, and 4), were preceding (initiating) normal lightning events. CIDs with field waveforms of types 7 and 11 were rarely isolated (18% and 15%, respectively) and most often were embedded in normal lightning activity. CIDs whose electric field waveforms exhibit ringing on the opposite-polarity overshoot are mostly isolated, while those without ringing in the electric field waveforms tend to initiate normal lightning events.

It is possible that types 7-11 CID waveforms are due to processes that are different from the "classical" CID process (which is still a subject of debate), but they are also different from "normal" IC-pulse waveforms (e.g., Nag *et al.*[2]). Note that types 7-11 are rare, and their exclusion from the data set would not materially influence the results presented in this Appendix.

References

1. Leal, A. F. R., Rakov, V. A. & Rocha, B. R. P. Compact intracloud discharges: New classification of field waveforms and identification by lightning locating systems. *Electr. Power Syst. Res.* **173**, 251–262 (2019).

2. Nag, A., DeCarlo, B. A. & Rakov, V. A. Analysis of microsecond- and submicrosecond-scale electric field pulses produced by cloud and ground lightning discharges. *Atmos. Res.* **91**, 316–325 (2009).
